# Function of Cyclophilin1 as a long-distance signal molecule in the phloem of tomato plants

**DOI:** 10.1093/jxb/erw487

**Published:** 2017-01-04

**Authors:** Ziv Spiegelman, Sumita Omer, Ben N. Mansfeld, Shmuel Wolf

**Affiliations:** 1 The Robert H. Smith Institute of Plant Sciences and Genetics in Agriculture, The Hebrew University of Jerusalem, The Robert H. Smith Faculty of Agriculture, Food and Environment, Rehovot 76100, Israel

**Keywords:** Auxin, cyclophilin, *diageotropica*, phloem, root development, *Solanum lycopersicum*.

## Abstract

Tomato (*Solanum lycopersicum*) *diageotropica* (*dgt*) mutants, containing a single mutation in the *Cyclophilin1* (*SlCyp1*) gene, are auxin-insensitive, exhibiting a pleiotropic phenotype including lack of geotropism, abnormal xylem structure, lack of lateral roots (LRs), and elevated shoot-to-root ratio. SlCyp1 is a putative peptidyl-prolyl isomerase that can traffic from shoot to root, where it induces changes in auxin response, LR formation, and xylem development, suggesting it has a role as a long-distance signaling molecule. Here, we explored the mechanism underlying SlCyp1 function in the phloem. Expression of *SlCyp1* under a phloem-specific (*AtSuc2*) promoter in *dgt* plants partially restored the wild-type phenotype, including lateral root development, root branching, and xylem morphology. The observed developmental changes were associated with physiological alternations at the whole-plant level, including a reduction in shoot-to-root ratio, enhanced transpiration, and elevated photosynthetic rates. Conversely, phloem-specific expression of *SlCyp1* active-site mutants did not restore the wild-type phenotype. Local inhibition of cyclophilin functioning in the target tissue reduced auxin sensitivity, suggesting that its enzymatic activity in the distant organ is required for its action as a long-distance signalling agent. The data presented suggest that SlCyp1 is a signal molecule trafficking from shoot to root where its activity is required for auxin-mediated lateral root development.

## Highlight

Phloem-specific expression and peptidyl-prolyl isomerase (PPI) activity are required for Cyclophilin1 action as a long-distance signal molecule, and auxin response modulation depends on activity of the trafficking protein in the target tissue.

## Introduction

Virtually all aspects of plant growth and development are dominated by auxins, a class of growth regulators (Woodward and Bartel, 2005). In roots, auxins control numerous developmental processes including cell division and elongation, cellular differentiation, root hair formation, and lateral root (LR) development (Teale *et al.*, 2006; Overvoorde *et al.*, 2010). The tomato (*Solanum lycopersicum*) *diageotropica* (*dgt*) mutant is auxin-insensitive, characterized by a pleiotropic phenotype including lack of geotropism, hyponastic leaves, impaired secondary growth, malformed secondary xylem vessels, short roots, and lack of LRs (Zobel, 1973, 1974). Several physiological studies have established that while the *dgt* mutant is not impaired in IAA synthesis (Fujino *et al.*, 1988) or uptake (Daniel *et al.*, 1989; Muday *et al.*, 1995), it is unresponsive to auxin (Kelly and Bradford, 1986) and exhibits a partial reduction in polar auxin transport (Ivanchenko *et al.*, 2015). Corresponding to the auxin-insensitive phenotype, the *dgt* mutation is associated with a reduction in the levels of auxin-responsive transcripts, including *small-auxin-up-regulated-RNA* (*SAUR*) (Mito and Bennett, 1995), *Aux/IAA*, and *ACC synthase* (Nebenführ *et al.*, 2000; Balbi and Lomax, 2003). Recent morphological studies have established that the absence of LRs in *dgt* mutants is due to an inability to form LR primordia from dividing pericycle cells (Ivanchenko *et al.*, 2006). A more recent study indicated that *dgt* mutant root caps contain elevated levels of hydrogen peroxide (H_2_O_2_), suggesting that this mutation inhibits the auxin response by modulating reactive oxygen species (ROS) levels at the root tip (Ivanchenko *et al.*, 2013).

Mapping of the *dgt* mutation established its location in the tomato *Cyclophilin1* (*SlCyp1*) gene (Oh *et al.*, 2006). Cyclophilins are chaperones that catalyze protein folding by peptidyl-prolyl cis-trans isomerase (PPI) activity and are involved in various cellular signaling pathways in different organisms (Wang and Heitman, 2005). Research conducted over the last few decades has demonstrated the involvement of cyclophilins in numerous biological processes. These include metabolic responses to oxidative stress and light intensity (Dominguez-Solis *et al.*, 2008), abiotic stress tolerance (Ruan *et al.*, 2011), plant–pathogen interactions (Deng *et al.*, 1998), and control over plant development and phase change (Berardini *et al.*, 2001). SlCyp1 orthologue mutants have been discovered in the moss *Physcomitrella patens* (Lavy *et al.*, 2012) and rice (Kang *et al.*, 2013; Zheng *et al.*, 2013), exhibiting phenotypes similar to those of the tomato *dgt*, including auxin insensitivity (Lavy *et al.*, 2012) and lack of LR formation (Kang *et al.*, 2013; Zheng *et al.*, 2013), suggesting that *Cyp1* has a conserved role in the auxin response pathway across the plant kingdom. Recent insights into the biochemical mechanism underlying the role of cyclophilins in auxin response were provided by studying the Cyp1 orthologue from rice, LATERAL ROOTLESS 2 (LRT2) (Jing *et al.*, 2015). The authors concluded that LRT2 directly interacts with the rice Aux/IAA protein, OsIAA11. This interaction further leads to the isomerization of the OsIAA11 proline peptide bond C^105^T^106^, which destabilizes OsIAA11 and thereby promotes the auxin signal.

Cyclophilin activity has been extensively studied in humans. This is mostly due to the fact that human Cyclophilin A (HsCypA) was identified as the target of the immunosuppressive drug Cyclosporin A (CsA) (Handschumacher *et al.*, 1984), which inhibits its PPI activity (Takahashi *et al.*, 1989). Site-directed mutagenesis studies on *HsCypA* defined the residues required for either PPI activity or the CsA binding affinity of the protein (Zydowsky *et al.*, 1992). Mutations in two active site residues, R55A and H126A, resulted in only 1% of the wild-type PPI activity, leaving the CsA binding affinity undisturbed. However, the W121A mutation retained 8.7% of the catalytic function, but CsA affinity was reduced up to 400-fold. The W121 residue was also found to be the major determinant of CsA binding affinity in several other studies (Liu *et al.*, 1991; Dorfman *et al.*, 1997).

It is now evident that the phloem translocation stream contains a distinct population of macromolecules, including mRNAs and proteins, that possess putative functions as long-distance signaling agents (Turgeon and Wolf, 2009; Ham and Lucas, 2013; Spiegelman *et al.*, 2013). Interestingly, cyclophilins have been found to be prominent in the phloem translocation stream. Members of this protein family have been detected in the phloem sap of several monocots and dicots species, including *Triticum aestivum*, *Oryza sativa*, *Ricinus communis*, *Cucurbita maxima*, *Lupinus albus*, and *Brassica oleracea* (Schobert *et al.*, 1998; Barnes *et al.*, 2004; Aki *et al.*, 2008; Lin *et al.*, 2009; Rodriguez-Medina *et al.*, 2011; Anstead *et al.*, 2013). RcCyp1, isolated from the phloem sap of *Ricinus communis*, was found to possess biochemical PPI activity, and mediate its own cell-to-cell movement (Gottschalk *et al.*, 2008).

We have recently established that the SlCyp1 protein can traffic long-distance through the phloem translocation stream from the shoot to the root, where it induces changes in xylem morphology, LR development, auxin response, and shoot-to-root ratio (Spiegelman *et al.*, 2015). While these findings suggest that SlCyp1 acts as a long-distance signal, additional evidence is required to establish the specific role this protein plays in the phloem, and whether its activity in the target site is required for its evident role in inter-organ communication.

In the current study, we further examined the specific role that SlCyp1 plays in the phloem of tomato plants. Expression of the wild-type *SlCyp1* gene in the phloem of the *dgt* mutant background resulted in partial restoration of the wild-type phenotype, including rescue of secondary xylem and LR development, an increase in root branching, and a decrease in shoot-to-root ratio. The observed developmental changes probably occurred through an increase in auxin sensitivity. Site-directed mutations indicated that PPI activity is essential for SlCyp1 activity in the phloem, and grafting experiments showed that Cyp1 expression in the shoot phloem is sufficient to rescue the root phenotype. Importantly, application of CsA to the rootstock only reduced the recovered auxin sensitivity following scion-to-rootstock trafficking of SlCyp1. These results indicate that, in addition to its long-distance trafficking, activity of the trafficking protein in the target tissue is required to exert developmental changes.

## Materials and methods

### Plant material and grafting protocol

Tomato (*Solanum lycopersicum*) *diageotropica* (*dgt*) mutants and their respective control *VFN8* plants were obtained from the Tomato Genetics Resource Center (TGRC) at UC Davis. Plants were grown in a soil mixture in 10-cm diameter plastic pots. Growth chamber conditions were a temperature of 25 ± 2/18 ± 2 °C day/night, and light intensity of 180 µmol m^–2^ s^–1^. Photoperiod was set at 12 h. For western blot experiments, different plant organs were collected from *Cucurbita maxima* (cv. Tripoli), *Cucumis sativus* (cv. Beit Alfa). and *Solanum lycopersicum* (cv. M82) at 4 weeks after sowing. For the different physiological measurements performed on *dgt*, *VFN8*, and *SlCyp1-PX* transgenic *dgt* lines, samples were collected 1 month after sowing. Reciprocal grafting experiments were performed with 4-week-old chamber-grown *dgt* and *SlCyp1-PX* plants using 0.6-mm wide plastic grafting tubes. Graftings were performed ~0.5cm under the cotyledons, resulting in source–sink relations between the scion and the rootstock, respectively. Measurements were taken 4 weeks after the time of grafting.

### Gravitropic response assay

Seeds were placed on moist Whatman paper in the dark at room temperature. When root length reached 2–5 mm, the intact seedlings were transferred to 10 × 10 cm 1% agar plates containing Nitch medium supplemented with vitamins (Duchefa Biochemie). Seedlings were placed horizontal to the gravity vector and grown in the same orientation for 48 h, after which the angles of curvature were measured.

### Cloning, site-directed mutagenesis, and plant transformation

To generate transgenic tomato plants expressing *SlCyp1* specifically in the phloem (*SlCyp1-PX*, where PX stands for phloem-expression), the *SlCyp1* open reading frame (ORF) was amplified from total cDNA of a cv. M82 leaf using extension primers adding a 6xHis tag sequence at the 5′ of the Cyp1 ORF, and PstI and HindIII sites at the 5′ and 3′, respectively (for primers, see Table 1). The PCR product was cloned downstream to the *Arabidopsis thaliana Suc2* promoter sequence (Truernit and Sauer, 1995) and upstream to the OCS terminator in the *pBJ36-pSuc2* vector (kindly supplied by Yuval Eshed, Weizmann Institute of Science, Israel), forming *pBJ36-pSuc2:6xHis-SlCyp1*. To generate the active-site SlCyp1 mutants, site-directed mutagenesis was performed on the *pBJ36-pSuc2:6xHis-SlCyp1* plasmid using the QuikChange Site-Directed Mutagenesis Kit (Agilent) according to the manufacturer’s protocol. Sense and antisense primers (Table 1) were designed to introduce the mutations in each of the targeted amino acids: *R62A*, *H133Q*, and *W128A*. The *pSuc2:6xHis-SlCyp1:OCS*_*ter*_ segment, as well as the mutant segments, were then cloned into the binary vector *pART27* (Gleave, 1992) using two *NotI* restriction sites. The binary plasmids were then transformed into *Agrobacterium tumerfaciens*, GV3101 strain. Cotyledon transformation was performed to tomato *dgt* mutants according to McCormick (1991).

**Table 1.** Primers used for cloning of *pSuc2:6xHis-Cyp1*, real-time PCR experiments and site-directed mutagenesis. Bold letters indicate the corresponding amino acid codon changed.

Gene	Forward	Reverse
*pSuc2:6xHis-Cyp1*	CAAATACGTGAAGGTAGCAGTTGAC	ACACCATTTGTAAGGTCCATAAGCT
*IAA10*	GACTTCTCAAAAGCTTGATCGAGAG	TGAAATCTTTCATTCCTTGGACAA
*IAA11*	AAAGAACAGTTTTAACGGACGTGAA	GACTTATCTGCATCCTCCAATGCT
*Tubulin*	GAAAGCCTACCATGAGCAGC	CTTTGGCACAACATCACCAC
R62A	GGCTCAACCTTCCAC**GCT**GTGATCCCAGGGTT	AACCCTGGGATCAC**AGC**GTGGAAGGTTGAGCC
H133Q	GGCTCAACGGAAAG**CAA**GTCGTGTTTGGACAAG	CTTGTCCAAACACGAC**TTG**CTTTCCGTTGAGCC
W128A	GTACCGCTAAGACTGA**GGC**GCTCAACGGAAAGCACG	CGTGCTTTCCGTTGAGC**GCC**TCAGTCTTAGCGGTAC

### Protein extraction and western blot analysis

Tissue samples of ~200 mg were taken from different plant organs. Samples were ground in liquid nitrogen using a mortar and pestle and then suspended in 500 µl of X4 protein sample buffer (40% glycerol: 8% SDS: 4% β-mercaptoethanol, pH6.8). Samples were centrifuged (4 °C, 10 000*g*) and supernatants were collected for protein analysis. Protein extracts were further separated by SDS-PAGE with 15% acrylamide in a Bio-Rad mini-gel system and electroblotted. Blots were blocked and incubated with 1:1000 diluted rabbit polyclonal antibodies directed against *A. thaliana* cyclophilin AtCYP18-3 (kindly provided by Charles Gasser, UC Davis) in 5% milk–TBS solution. Horseradish peroxidase conjugated goat anti-rabbit antibody (Sigma-Aldrich, http://www.sigmaaldrich.com) was used as the secondary antibody (dilution 1:50 000). Detection was performed by film exposure following ECL (Enhanced Chemilluminescence System, Thermo Scientific, http://www.thermoscientific.com) incubation.

### Light microscopy

Stem samples were collected ~2.5 cm below the cotyledons and root samples were collected ~7 cm above the root cap. Samples were harvested and trimmed to 5 mm length, and were then fixed in FAA solution (10% formaldehyde: 5% acetic acid: 85% ethanol), subjected to vacuum for 30 min, and dehydrated at room temperature in a graded ethanol series (30 min each at 50, 70, 90, 95, and 100%). Samples were infiltrated and embedded in paraffin according to Ruzin (1999). Paraffin-embedded tissue was then cut by microtome (Leica RM2245) into 15–18-µm sections and transferred to microscope slides. Slides were deparaffinized twice for 10 min each in 100% Histocler (Gadot, http://www.gadot.com), followed by one wash in 100% ethanol (2 min), and then were air-dried under the chemical fume hood for 5 min. Sections were stained with safranin and fast green dye (Ruzin, 1999). The sections were observed with a light microscope (Olympus BX50, ×50–100 magnifications).

### Immunolocalization

SlCyp1 immunolocaliztion was performed as previously described by Paciorek *et al.* (2006), with the following modifications. Stem samples were collected from *VFN8* plants below the cotyledons, and trimmed to 5 mm length. Samples were then fixed in a paraformaldehyde solution (4% formaldehyde, 5% acetic acid in 1×PBS), infiltrated and embedded in Wax (90% PEG, 10% 1-hexadecanol) and then sectioned (15 μm thick) in a cooled microtome (Leica RM2245). Sections were transferred to microscope slides for incubation with 1:100-diluted rabbit polyclonal antibodies directed against *A. thaliana* cyclophilin AtCYP18-3 (1×PBS containing 2% BSA). Fluorescein isothiocyanate (FITC) -conjugated goat anti-rabbit (Sigma-Aldrich) was used as the secondary antibody (dilution 1:50).

### Confocal microscopy

Observations and acquisition of images were performed with an Olympus IX-81 confocal laser scanning microscope (CLSM; FV 500, Olympus Optical Co., Tokyo, Japan) equipped with a 405-nm diode laser, 488-nm argon-ion laser, and a UPlanApo 20× NA 0.7 objective. Cell walls were excited by 405 nm light and the emission was collected through a BA 430–460 filter. FITC was excited by 488 light and emissions collected through BA 515–525 barrier filter.

### IAA and CsA response assays

Stem segments were excised from the different rootstocks at 3 weeks after grafting. Segments were taken from 1.5–5 cm below the graft union. These sections were cut to short pieces of about 0.5 cm and incubated in a MES-sucrose solution [10 mM MES buffer, 1% (w/v) sucrose, pH 6.0] for 2 h to deplete endogenous auxin. Segments were then transferred to a 100-mM sodium potassium citrate buffer (pH 4.6) (control) or buffer containing either 0.1 mM of indole-3-acetic acid (IAA) or 0.1 mM IAA + 10 µM cyclosporin A (CsA). Samples were gently shaken for 2 h at room temperature. After incubation, they were soaked and immediately frozen in liquid nitrogen and stored at –80°C for later analysis.

### RNA isolation and quantitative RT-PCR

Samples of stem segments 5-cm long were taken 0.5 cm above the root–shoot junction from plants of *dgt*, *SlCyp1-PX* lines 2 and 9, and *VFN8*. Samples were frozen in liquid nitrogen and stored at –80 °C until use. Total RNA was extracted from 500 mg of the samples, using Tri-reagent (Sigma-Aldrich, http://sigmaaldrich.com) according to the manufacturer’s protocol. RNA was quantified by NanoDrop 2000C analyser (ThermoFisher Scientific, http://thermofisher.com). cDNA was prepared from the same amounts of RNA (1 µg) per sample pretreated with 1 unit µg^–1^ of RQ1 DNAse (Promega, http://promega.com), using the Verso cDNA synthesis kit (ThermoFisher Scientific). Real-time RT-PCR reactions were carried out using 0.5 µl of 2.5 pmol of each primer (see Table 1), 4 µl cDNA, and 5 µl ABsolute^TM^ Blue QPCR SYBER^®^ Green ROX Mix. PCR conditions were 95 °C for 15 min (enzyme activation), and then the following cycle, repeated 45 times: 95 °C for 10 s, 59 °C for 15 s, and 72 °C for 25 s. The obtained cycle temperature (CT) values were analysed with Rotor-Gene 6000 Series software by averaging the two independently calculated normalized expression values of the duplicates. The calculated *IAA10* and *IAA11* numerical values were divided by the values obtained for the housekeeping gene *tubulin* in each respective sample (for primers, see Table 1).

### Gas exchange measurements

Gas exchange measurements were taken using the LI-6400 portable gas-exchange system (Li-Cor). Photosynthesis, CO_2_ concentrations in the substomatal cavities (*C*_i_), stomatal conductance, and transpiration rates were measured in young source leaves (4th leaf from the apex) of 6-week-old tomato plants. Plants were grown in an environmentally controlled greenhouse. Measurements were performed at around noon in a constant CO_2_ concentration of 390 ppm and constant photosynthetic photon flux density (PPFD) of 1200 µmol m^–2^ s^–1^. For PPFD response curve, plants were subjected to the following radiation levels: 200, 400, and 800 µmol m^–2^ s^–1^.

## Results

### Expression patterns of SlCyp1 in plants

Previous research has established that Cyp1 proteins are present within the phloem sap of different species (Schobert *et al.*, 1998; Gottschalk *et al.*, 2008) and that the *SlCyp1* gene is expressed in the tomato vasculature and lateral root primordia (Ivanchenko *et al.*, 2015). However, it is not clear if Cyp1 is selectively accumulated in the phloem sap or constitutively expressed in other plant tissues and secreted passively into the phloem translocation stream. To determine the organs in which Cyp1 is accumulated, expression pattern analyses were performed.

**Fig. 1.**
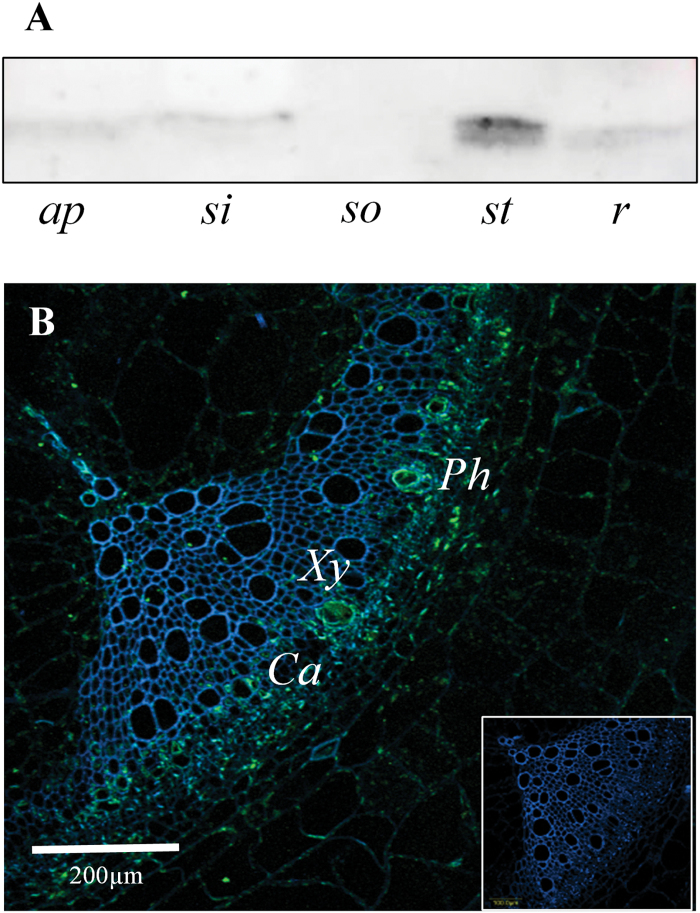
Spatial distribution of Cyp1 proteins in different organs. (A) Anti-cyclophilin western-blot analyses on protein extracts from different tomato organs using an anti-AtCyp18-3/ROC1 antiserum. Proteins were extracted from the following organs: shoot apex (*ap*), sink leaf (*si*), source leaf (*so*), stem (*st*), and root (*r*). (B) SlCyp1 immunolocalization in a transverse section of tomato stem. The green signal indicates SlCyp1 and the blue signal indicates xylem autofluorescence. Note that SlCyp1 localizes mainly to the phloem (*Ph*), cambium (*Ca*), and developing xylem (*Xy*) vessels. Inset: negative control. Images were obtained using an Olympus IX-81 confocal microscope.

In tomato, the highest accumulation of Cyp1 was evident in stems (Fig. 1A). A strong Cyp1 signal was obtained in the phloem sap and apices of cucurbit plants (see Supplementary Fig. S1). Cyp1 was also detected, to a lesser extent, in sink leaves, and lower levels of Cyp1 were observed in source leaves and in roots (Fig. 1A; Supplementary Fig. S1). While phloem sap can be easily collected from cucurbits, it cannot be obtained from tomatoes. Therefore, immunolocalization analysis was performed on tomato stem cross-sections (Fig. 1B). In agreement with previous results (Spiegelman *et al.*, 2015), this analysis indicated that SlCyp1 localizes mainly in areas peripheral to the xylem and phloem regions.

### Partial restoration of wild-type phenotype by companion-cell-specific expression of *SlCyp1* in *dgt* mutants

**Fig. 2.**
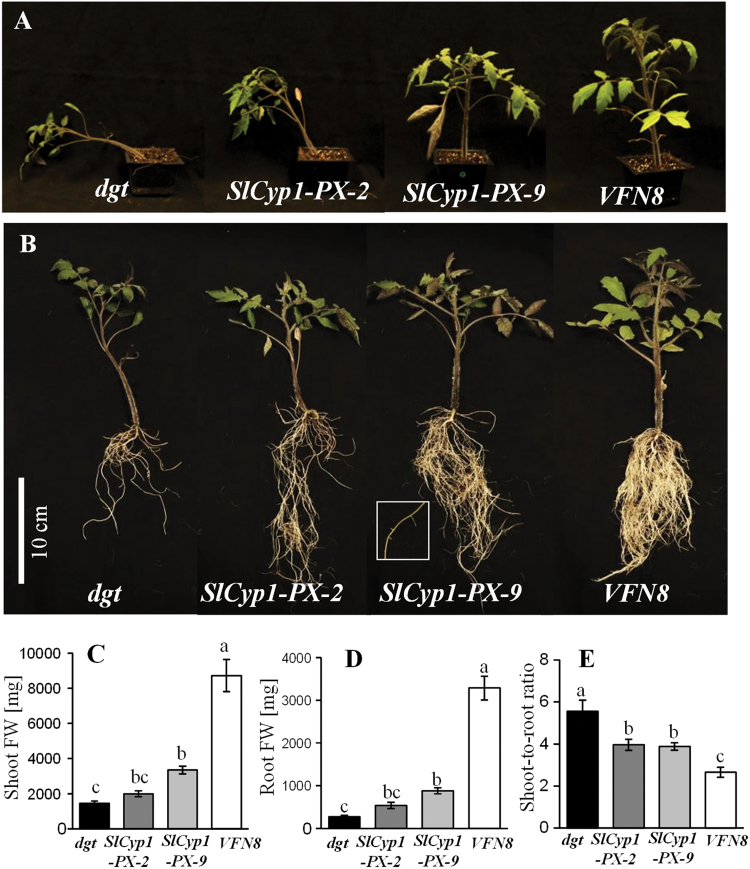
Effect of phloem-specific expression of *SlCyp1* on plant growth and development. Expression of *SlCyp1* under the *AtSuc2* promoter in *dgt* mutant lines (*SlCyp1-PX* lines) results in phenotypic recovery. The images show shoots (A) and roots (B) of 4-week-old plants. Data for shoot fresh weight (C), root fresh weight (D), and shoot-to-root ratio (E) of *dgt* mutants (black bars), *SlCyp1-PX-2* (dark grey bars), *SlCyp1-PX-9* (light grey bars), and *VFN8* control plants (white bars). Data represent means of 10 replications (±SE). Identical letters indicate no significant differences between genotypes at *P*<0.05 by Tukey’s HSD-test. (This figure is available in colour at *JXB* online.)

To further explore the functional role of SlCyp1 in the phloem, *dgt* mutants were transformed with a *6xHis*-tagged *SlCyp1* gene under the companion-cell-specific *AtSuc2* promoter from Arabidopsis (*SlCyp1-PX* plants). This promoter was also found to be phloem-specific in tomato plants (Golan *et al.*, 2013). Out of five independent transgenic *SlCyp1-PX dgt* lines, two were characterized as representatives: *SlCyp1-PX*-2 and *SlCyp1-PX*-9, exhibiting low and high levels of the *6xHis-SlCyp1* transcripts, respectively (see Supplementary Fig. S2). Interestingly, while the *dgt* mutants exhibited a typical droopy stature, *SlCyp1-PX* plants were characterized by partial restoration of the wild-type phenotype, similar to that of *VFN8* control plants (Fig. 2A). Moreover, the *SlCyp1-PX* plants had more shoot biomass (Fig. 2C) and were taller than the *dgt* mutants (Supplementary Fig. S2). In addition, *SlCyp1-PX* plants had wider stems than the *dgt* plants, as indicated by their diameter (Supplementary Fig. S2).

One of the most prominent characteristics of the *dgt* mutant is impaired root growth and lack of LRs (Fig. 2B). Interestingly, the transgenic *SlCyp1-PX* plants had significantly higher root volume and length (Fig. 2B; Supplementary Fig. S2) than the *dgt* mutants, but not to the level of *VFN8* plants. This increase in root volume was due to induction of LR development (Fig. 2B). The enhanced root growth and branching led to an increase in root biomass (Fig. 2D), as compared to that of the *dgt* mutants. Interestingly, the enhanced root growth and branching in *SlCyp1-PX* plants led to a lower shoot-to-root ratio than that of *dgt* mutants (Fig. 2E). These results suggest that phloem expression of *SlCyp1* may be required to balance root growth with that of the shoot, leading to a controlled shoot-to-root ratio.

Notably, restoration of the wild-type phenotype in the plant line *SlCyp1-PX-9* was stronger than in the line *SlCyp1-PX-2*. This suggests that the rescue of the *dgt* mutant phenotype is a result of the level of *SlCyp1* expression in the phloem (see Supplementary Fig. S2).

### Phloem-specific expression of *SlCyp1* promotes xylem development, water transport, and photosynthetic activity

**Fig. 3.**
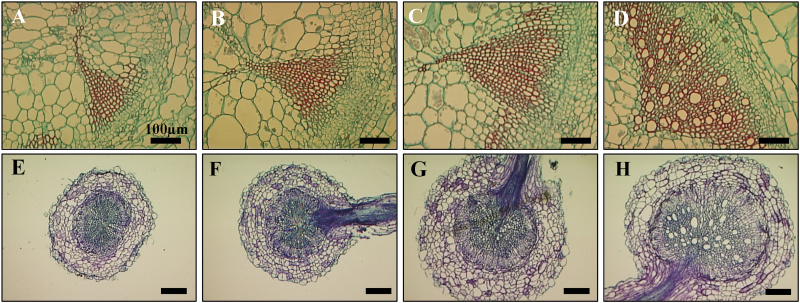
Ultrastructural changes in stems and roots in *dgt*, control, and transgenic plants expressing *SlCyp1* in the phloem. Transverse sections of stems (A–D) and roots (E–H) in *dgt* mutants (A, E), *SlCyp1-PX-2* (B, F), *SlCyp1-PX-9* (C, G), and *VFN8* control plants (D, H). Stems of *dgt* mutants exhibit small numbers of narrow and fibrous xylem vessels (A, E) compared to *VFN8* control plants (D, H). Note the lateral-root sites of origin (F–H). Stem sections were excised ~3 cm below the cotyledons. Root sections were excised ~2 cm below the shoot–root junction. Scale bars = 100 µm.

An additional set of experiments was aimed at studying the effect of phloem-specific *SlCyp1* expression on xylem development. Transverse sections showed that the *dgt* mutants are characterized by a severely impaired xylem system, with the number of vessels being reduced, and those that are present being more narrow and fibrous (Fig. 3A and E) compared with the wide xylem vessels of the *VFN8* control plants (Fig. 3D and H) (see also Spiegelman *et al.*, 2015). Phloem-specific expression of *SlCyp1* resulted in partial rescue of xylem development in both the *SlCyp1-PX* lines (Fig. 3B, C and F, G), which exhibited an increased number of xylem vessels that were slightly wider than those of the *dgt* mutants (Fig. 3A, E). In addition to restoration of xylem development, the *SlCyp1-PX* lines were characterized by LR formation (Fig. 3F, G), similar to that of the *VFN8* control plants (Fig. 3H).

**Fig. 4.**
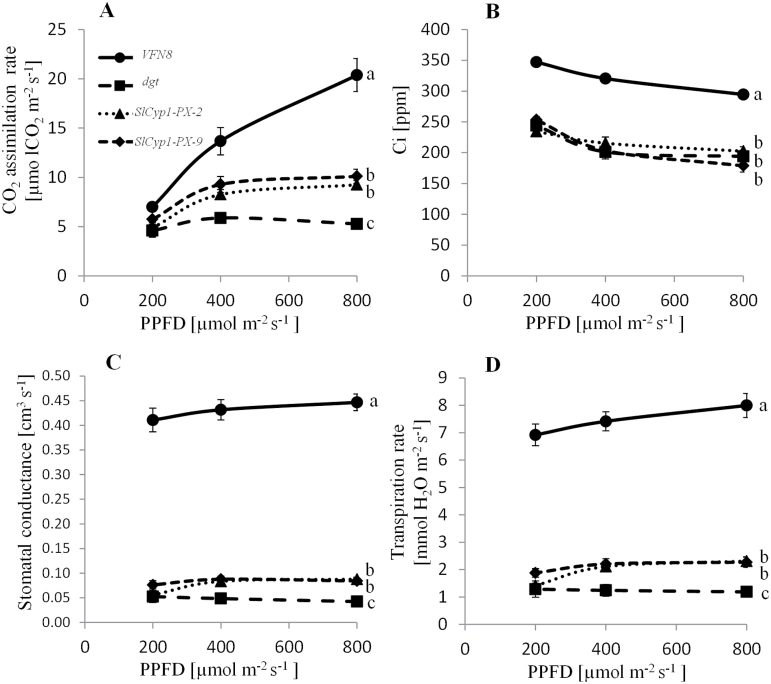
Effect of phloem-specific expression of *SlCyp1* on photosynthesis and leaf gas exchange parameters. Data for photosynthesis (A), CO_2_ concentrations in the substomatal cavities (*C*_i_) (B), stomatal conductance (C), and transpiration rate (D) of *dgt* mutants, *VFN8* control plants, *SlCyp1-PX-2*, and *SlCyp1-PX-9* are presented. Data represent means of four biological replications (±SE). Identical letters indicate no significant differences between genotypes at *P*<0.05 by Tukey’s HSD-test.

It is logical to assume that the lack of lateral root and aberrant xylem vessels in the *dgt* mutant leads to water deficit that further affects transpiration rate and photosynthetic activity. As anticipated, the photosynthetic rate of the *dgt* mutants was lower than that of *VFN8* control plants, with limited response to increasing photosynthetic photon flux density (PPFD) (Fig. 4A). In concordance with the developmental recovery, photosynthetic rates of *SlCyp1-PX* plants were higher than those of *dgt* mutants (Fig. 4A). Interestingly, while CO_2_ concentrations in the substomatal cavities (*C*_i_) were similar in the *SlCyp1-PX* plants and *dgt* mutants (Fig. 4B), stomatal conductance and transpiration rates were significantly higher in *SlCyp1-PX* plants (Fig. 4C, D). These results suggest that the reduced photosynthetic activity in the *dgt* mutants is not due to impairment in the carboxylation activity, as CO_2_ homeostasis at the substomatal cavities is not disturbed. The deficient photosynthesis can rather be explained by lower CO_2_ diffusion as a result of stomatal closure, which is associated with inhibited transpiration and root activity.

### Restoration of auxin response by phloem-specific expression of SlCyp1 depends on its PPI active site

**Fig. 5.**
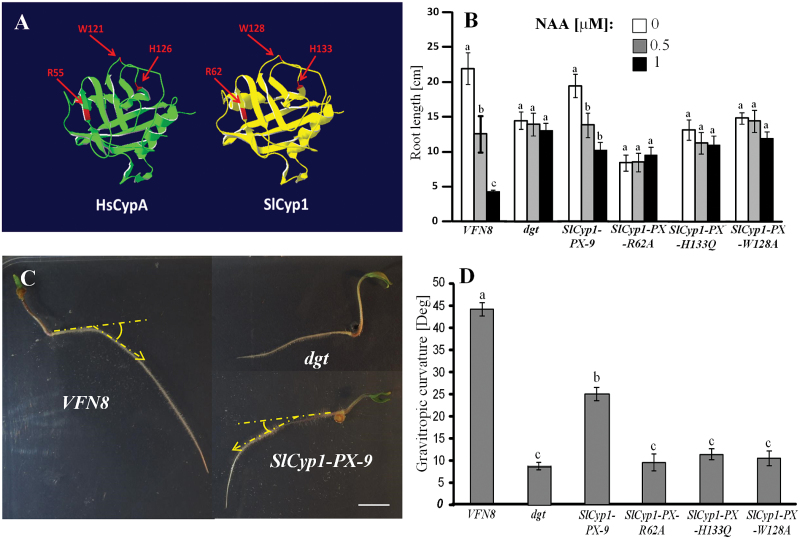
The PPI active site is required for SlCyp1 function in the phloem to control auxin response. (A) 3D structure of HsCypA (left) and a homology-based model of SlCyp1 (right). The shaded areas at the end of the arrows indicate the active-site residues that were targeted for mutagenesis in HsCypA (Zydowsky *et al.*, 1992) and the corresponding amino-acids in SlCyp1. 3D modeling was performed according to Arnold *et al.* (2006). (B) Primary root length of *VFN8*, *dgt*, *SlCyp1-PX-9*, *SlCyp1-PX-R62A*, *SlCyp1-PX-H133Q*, and *SlCyp1-PX-W128A* tomato seedlings grown on various concentrations of NAA: 0 µM (white bars), 0.5 µM (grey bars), or 1 µM (black bars). Tomato seeds were sown on standard germination paper soaked with distilled water containing each of the indicated NAA concentrations. Root length measurements were taken 14 d after sowing. (C) Images showing the gravitropic response of *VFN8*, *dgt*, and *SlCyp1-PX-9* seedlings 48 h after a gravity stimulus. Soon after the emergence of the radicle (2–5 mm) seedlings were oriented horizontal to the gravity axis (represented by the dashed lines) and allowed to grow for 48 h, after which the angle of curvature (represented by the dashed arrows) was measured. Scale bar = 1 cm. (D) Gravitropic curvature angles of *VFN8*, *dgt*, *SlCyp1-PX-9*, *SlCyp1-PX-R62A*, *SlCyp1-PX-H133Q*, and *SlCyp1-PX-W128A* 48 h after the gravity stimulus. The data represent the means of six biological replications (±SE). Identical letters indicate no significant differences between each replicate at *P*<0.05 by Tukey’s HSD-test. (This figure is available in colour at *JXB* online.)

The pleiotropic phenotype of the *dgt* mutants is caused by the lack of auxin sensitivity (Zobel, 1973; Kelly and Bradford, 1986). It is therefore hypothesized that the PPI activity of the phloem-expressed SlCyp1 plays a role in modulating auxin response. To test this hypothesis, we first identified the amino acid residues that are important for SlCyp1 biochemical function. Previous mutational analysis of the human HsCypA established that substituting histidine 126 with glutamine (H126) or arginine 55 with alanine (R55A) result in a >99% loss of PPI activity (Zydowsky *et al.*, 1992; Chatterji *et al.*, 2009). The tryptophan 121 to alanine mutation (W121A) resulted in an 80- to 400-fold reduction in cyclosporine binding affinity and a 92% reduction in PPI activity (Bossard *et al.*, 1991; Zydowsky *et al.*, 1992). To determine the analogous residues in SlCyp1, we used a combination of protein sequence alignment (see Supplementary Fig. S3) and 3D homology modeling (Fig. 5A) with HsCypA. Notably, the identified active-site residues in HsCypA are conserved in SlCyp1: residues R62, W128, and H133 in SlCyp1 are analogous to HsCypA R55, W121, and H126, respectively (Fig. 5A; Supplementary Fig. S3). We further introduced these mutations to *SlCyp1* using site-directed mutagenesis, and generated transgenic *dgt* mutants expressing each of the mutated genes under the *AtSuc2* promoter. The lines were named according to the specific mutation: *SlCyp1-PX-R62A*, *SlCyp1-PX-W128A*, and *SlCyp1-PX-H133Q*.

To determine auxin response capacity, the *dgt* mutants, the various transgenic lines, and the *VFN8* control plants were germinated on different concentrations of naphthalene acetic acid (NAA) (Fig 5B). While the response of the *VFN8* control plants to NAA was dramatic and resulted in clear restriction of root elongation, the *dgt* mutants did not respond to NAA application and root length remained similar at all NAA concentrations. *SlCyp1-PX*-9 plants exhibited partial restoration of the response to auxin, resulting in a significant inhibition of root elongation under higher NAA concentrations (Fig 5B); however, none of the three *SlCyp1* active-site mutants responded to NAA. The restored auxin response of plants expressing the native SlCyp1 (*SlCyp1-PX*-9) is in agreement with the transcription levels of *IAA10*, an auxin- responsive *Aux/IAA* gene, which were significantly higher in *SlCyp1-PX-9* and *SlCyp1-PX-2* plants than in *dgt* mutants (see Supplementary Fig. S4).

Another process indicative of auxin activity is gravitropic response. We tested gravitropic response in *SlCyp1-PX-9* plants and in *dgt* plants expressing the mutated SlCyp1. Here, minimal gravitropic response was observed in *SlCyp1-PX-R62A*, *SlCyp1-PX-W128A*, and *SlCyp1-PX-H133Q* plants, similar to that of the *dgt* mutants (Fig. 5D). Partial gravitropism recovery was observed in *SlCyp1-PX*-9 seedlings as compared to the *VFN8* control plant (Fig 5C, D). Collectively, these results suggest that SlCyp1 biochemical activity in the phloem plays an important role in controlling auxin activity.

### Phloem-expression of *SlCyp1* in the shoot is sufficient to restore root growth and shoot-to-root ratio in *dgt* mutants

**Fig. 6.**
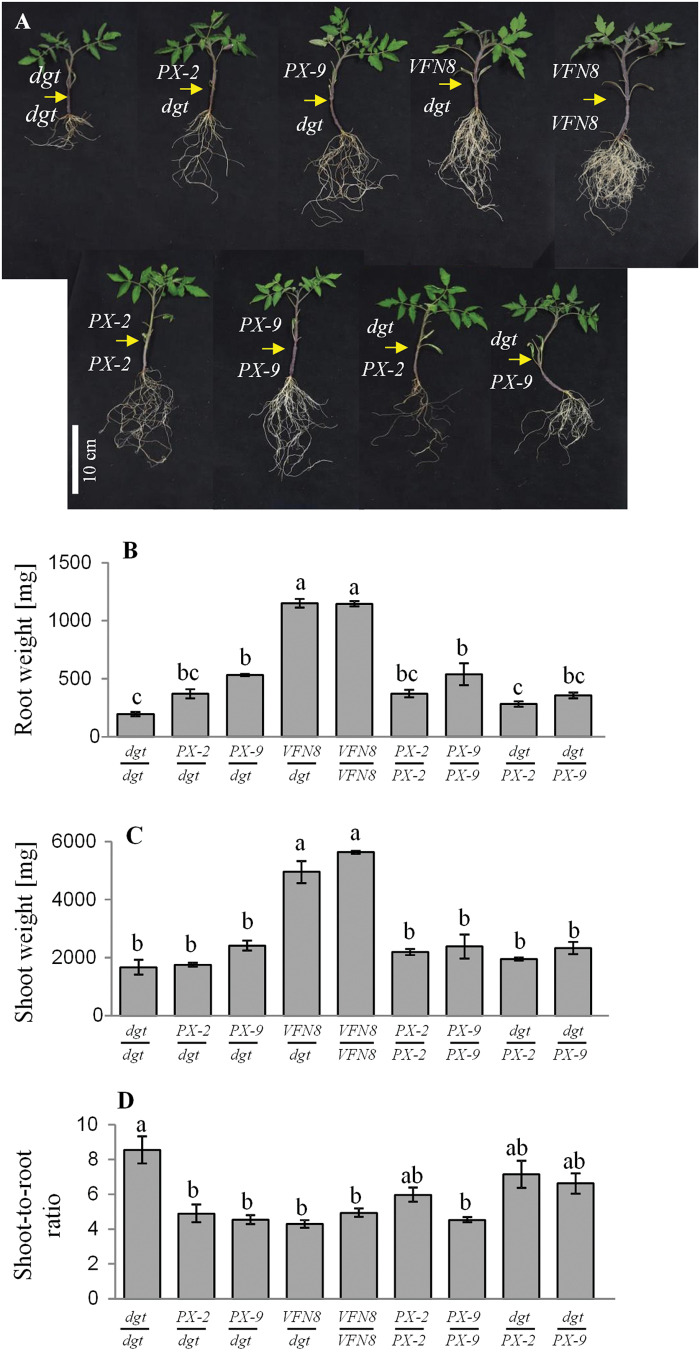
Reciprocal graftings of *SlCyp1* phloem-expressing plants with *dgt* mutants affect root development and shoot-to-root ratio. Reciprocal grafting experiment using *dgt* mutants grafted with *SlCyp1-PX-2* (*PX-2*), *SlCyp1-PX-9* (*PX-9*), and *VFN8* plants. (A) Images showing each of the different reciprocal grafts, as indicated. The arrows mark the grafting point in each plant. Root weight (B), shoot weight (C), and shoot-to-root ratio (D) were measured 4 weeks after grafting. The data represent the means of five biological replications (±SE). Identical letters indicate no significant differences between each graft at *P*<0.05 by Tukey’s HSD-test. (This figure is available in colour at *JXB* online.)

Grafting experiments have established that long-distance trafficking of the SlCyp1 protein from the *VFN8* scion to the *dgt* rootstock restored auxin response and development of lateral roots (Spiegelman *et al.*, 2015). To determine if exclusive expression of SlCyp1 in the shoot phloem can rescue the *dgt* root phenotype, grafting experiments were performed using *dgt*, *SlCyp1-PX-9*, *SlCyp1-PX-2*, and *VFN8* control plants. *dgt* rootstocks grafted onto *dgt* scions had under-developed root systems and lacked LRs, as compared to homografted *SlCyp1-PX-9* and *VFN8* plants (Fig. 6A, B). In marked contrast, the shoot biomass of *SlCyp1-PX* homografted plants was not significantly different from that of *dgt* homografts (Fig. 6C). Consequently, the shoot-to-root ratio of the homografted *dgt* plants was significantly higher than that of the homogratferd *SlCyp1-PX* plants (Fig. 6D). Consistent with the previous results, when *VFN8* scions were grafted onto *dgt* rootstocks, a recovery in *dgt* root growth was evident (Fig. 6A), resulting in elevated root biomass (Fig. 6B). Of note here is that a recovery in *dgt* root system was also observed when *dgt* rootstocks were grafted onto *SlCyp1-PX* scions (Fig. 6A), leading to a significant increase in root biomass (Fig. 6B). In contrast with the differences in root biomass accumulation, no differences in shoot biomass were evident between the *SlCyp1-PX* scions grafted onto *dgt* rootstocks and homografted *dgt* scions (Fig. 6C). This resulted in reduced shoot-to-root ratios in *dgt* rootstocks grafted onto *SlCyp1-PX* scions compared to homografted *dgt* rootstocks (Fig. 6D). When *dgt* scions were grafted on *SlCyp1-PX* rootstocks, root weight was lower compared to homografted *SlCyp1-PX* plants (Fig. 6A, B), and the shoot-to-root ratio was not different from that of the homografted *dgt* mutants (Fig. 6D). These results indicated that phloem-specific expression of *SlCyp1* in the shoot is sufficient to activate a SlCyp1-derived long-distance signal to promote root growth and reduce shoot-to-root ratios. Conversely, when *SlCyp1* is absent from the shoot, a reduction in root growth is observed, causing elevated shoot-to-root ratio.

### Auxin response in hetereografted *dgt* rootstock is suppressed by CsA, a cyclophilin inhibitor

**Fig. 7.**
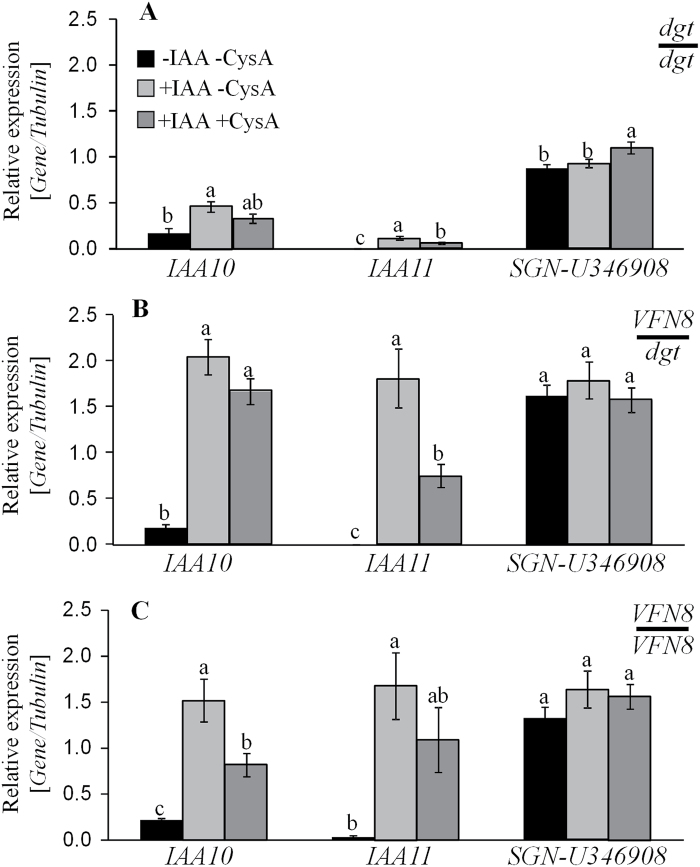
Cyclosporin A inhibits auxin-induced expression of *Aux/IAA* genes. Relative expression of *IAA10*, *IAA11*, and the *SGN-U346908* control gene determined in stem sections taken from rootstocks of the following scion/rootstock combinations: *dgt*/*dgt* (A), *VFN8*/*dgt* (B), and *VFN8*/*VFN8* (C). Transcription levels were measured following one of the indicated treatments: incubation in control solution (black bars), in a solution containing 0.1 mM IAA (light grey bars), or in a solution containing 0.1 mM IAA + 10 µM CysA (dark grey bars). Transcription levels were normalized using *tubulin* as an internal control. To verify that IAA or CysA does not have a general impact on gene expression *SGN-U346908*, which was characterized as stable housekeeping gene (Expósito-Rodríguez *et al.*, 2008), was selected as an internal control. Data represent means (±SE) (*n*=5 independent experiments). A Student’s *t*-test indicated a significant (*P*<0.05) effect of the chemical treatment on the expression levels within each grafting combination. Identical letters indicate no significant differences between each graft at *P*<0.05 by Tukey’s HSD-test.

Given that SlCyp1 is a long-distance trafficking protein (Spiegelman *et al.*, 2015), it is logical to assume that restoration of auxin sensitivity in *dgt* rootstocks grafted to *VFN8* or *SlCyp1-PX* tomato scions is a result of SlCyp1 trafficking to the heterografted *dgt* rootstock. However, as PPI activity is required for the functioning of the protein, restoration of auxin response may also be explained by trafficking of an additional molecule, which is subjected to the chaperonin activity of SlCyp1. To explore whether SlCyp1 activity is required in the target tissue (root) or only in the shoot, auxin response was tested in rootstock stems (1.5–5 cm below the graft union) from *VFN8*–*dgt* reciprocal graftings in the presence of the cyclophilin inhibitor CsA. Sections of rootstock stem were incubated in an IAA solution with or without the addition of CsA. In agreement with earlier findings (Nebenführ *et al.*, 2000; Spiegelman *et al.*, 2015), auxin treatment caused a significant increase in the expression of both *IAA10* and *IAA11* in homografted *VFN8* rootstock stems with minimal effect on expression of these genes in homografted *dgt* rootstocks (Fig. 7A, C). A significant increase in expression of the *Aux/IAA* transcripts was evident in *dgt* rootstocks grafted to *VFN8* scions (Fig. 7B). Application of CsA resulted in reduction of the IAA-induced up-regulation of both *IAA10* and *IAA11*. This reduction was statistically significant for the levels of *IAA10* in homografted *VFN8* rootstocks (Fig 7C) and for the levels of *IAA11* in *dgt* rootstocks grafted to *VFN8* scions (Fig. 7B). Levels of the control housekeeping gene, *SGN-U346908*, were similar under all IAA and CsA treatments, suggesting that CysA inhibited specifically the expression of auxin response genes (Fig. 7A–C). These results suggest that the restoration of auxin response in *dgt* rootstocks grafted to *VFN8* scions is attributable to the long-distance trafficking and activity of SlCyp1 at the target site.

## Discussion

Proteins from the cyclophilin family have been identified in the phloem sap of various species (Schobert *et al.*, 1998; Barnes *et al.*, 2004; Aki *et al.*, 2008; Lin *et al.*, 2009; Rodriguez-Medina *et al.*, 2011; Anstead *et al.*, 2013). However, their function in the sieve tube and in the phloem translocation stream has been unclear. More recent evidence has established that SlCyp1 long-distance trafficking from shoot to root can alter auxin response, xylem morphology, and root development (Spiegelman *et al.*, 2015). The current study focused on the specific activity of *SlCyp1* in the phloem, and its function in long-distance signaling. Phloem-specific expression of *SlCyp1* was sufficient to partially restore the normal phenotype in the auxin-insensitive *dgt* mutant. This includes rescue of shoot and root growth, LR development (Fig. 2), xylem-vessel formation (Fig. 3), and transpiration and photosynthetic rates (Fig. 4). Rescue of the *dgt* root phenotype could also be partially achieved by the grafting of *SlCyp1-PX* plants onto *dgt* mutant rootstocks. Moreover, inhibition of cyclophilin activity in the target tissue resulted in reduced auxin response. These observations are consistent with the idea that trafficking of SlCyp1 in the phloem, and not of a downstream molecule, acts as a shoot-to-root mobile signal (Spiegelman *et al.*, 2015).

Phloem-specific expression of *SlCyp1* in *dgt* plants did not fully restore the wild-type phenotype. One possible explanation can be attributed to the nature of the promoter. The *AtSuc2* promoter, which is a constitutive and CC-specific (Truernit and Sauer, 1995), may be weaker than the endogenous *SlCyp1* promoter. It should be taken into account that the endogenous *SlCyp1* promoter is not CC-specific and is also active in the surrounding cell layers (Ivanchenko *et al.*, 2015) from which SlCyp1 proteins could be loaded to the phloem. It is possible that the level of SlCyp1 accumulated in the sieve tube, when expressed under the *AtSuc2* promoter, is lower than that of wild-type tomato plants. Another explanation for the lack of full complementation is that *SlCyp1* is subjected to post-transcriptional regulation limiting its expression or activity. Nevertheless, the results presented here indicate that expression of SlCyp1 exclusively in the phloem is sufficient for its influence over developmental and physiological process in distant tissues.

Several studies have established that phloem-specific expression of auxin-responsive genes can modify root architecture. In Arabidopsis, phloem expression of the *IAA18* transcription factor negatively regulates LR formation (Notaguchi *et al.*, 2012). In tomato, phloem-specific expression of an *Aux/IAA* transcript, *F-308* (Omid *et al.*, 2007), resulted in a dramatic increase in the number of LRs and root biomass (Golan *et al.*, 2013). Auxin response in the phloem was found to be critical for LR development in maize (Jansen *et al.*, 2012), with an auxin-response maximum in the protophloem acting as a trigger preceding LR organogenesis, while abolishment of this phloem-specific auxin response resulted in random cellular divisions (Jansen *et al.*, 2012). Interestingly, uncontrolled cell division rather than the differentiation of LR primordia was also observed when *dgt* mutants were treated with auxin (Ivanchenko *et al.*, 2006). Taken together, the data suggest that the phloem plays an important role in linking auxin-induced differentiation with cell division. Our results, demonstrating that auxin response is affected by the accumulation of SlCyp1 in the phloem prior to LR differentiation, support this concept. It is important to note that SlCyp1 is expressed in the phloem of our transgenic *dgt* mutants, while LRs emerge from pericycle cells adjacent to xylem poles. This implies that either SlCyp1 or an unknown downstream signal move locally from the phloem to the sites of LR initiation. It is logical to assume that this signal provides an important connecting link between phloem activity and local auxin responses in LR formation.

PPI activity of various plant cyclophilins, including SlCyp1, has been demonstrated *in vitro* (Grebe *et al.*, 2000; Gottschalk *et al.*, 2008; Iki *et al.*, 2012; Kaur *et al.*, 2015); however, little is known about the biological significance of *in vivo* PPI activity in plants. Our results suggest that the conserved amino acid residues required for PPI activity of HsCypA (Zydowsky *et al.*, 1992) are also essential for SlCyp1 function in the tomato phloem. Interestingly, a recent study established that the rice orthologue of SlCyp1, LRT2, catalyzes the cis/trans isomerization of a proline peptide bond in OsIAA11 to promote its degradation and stimulate auxin response (Jing *et al.*, 2015). This result may point to a biochemical link between cyclophilin PPI activity and regulation of auxin signaling.

The observed recovery in transpiration and photosynthetic rates in *dgt* plants expressing SlCyp1 in the phloem can be largely attributed to the partial rescue of secondary xylem vessels and LR development. The formation of a branched root system and wider xylem vessels allowed improved water transport that in turn affected stomatal conductance and transpiration rates. Insights into the biological mechanism connecting auxin response, xylem-vessel diameter, and photosynthesis were provided by a study conducted on poplar trees subjected to salt stress. These trees exhibited a significant reduction in xylem-vessel diameter and lower transpiration rate that was associated with reduced concentration of free auxin in the xylem. Overexpression of the poplar auxin-amidohydrolase, ILL3, which releases active IAA from storage auxin conjugates, in Arabidopsis plants alleviated the sensitivity to salt stress (Junghans *et al.*, 2006). It can be hypothesized that SlCyp1 functions in the phloem to modulate auxin-mediated development of xylem vessels for the coordination of root water uptake with xylem water transport and transpiration.

Collectively, the research presented here established that *SlCyp1* plays a role in the tomato phloem to regulate several auxin-mediated developmental processes, including xylem development, LR formation, and root branching, that affect transpiration rate and photosynthetic activity. The functioning of SlCyp1 as a long-distance signal molecule depends on its PPI activity. Moreover, the trafficking protein must be active at the target sites in order to exert its influence on auxin response. Nevertheless, at this point we cannot rule out the possibility that SlCyp1 functions in the phloem as a complex with additional protein(s) that are potentially involved in the auxin response pathway.

## Supplementary data

Supplementary data are available at *JXB* online.

Fig. S1. Spatial distribution of Cyp1 proteins in different organs.

Fig. S2. Effect of phloem-specific expression of *SlCyp1* on shoot and root growth.

Fig. S3. Homology of the human CypA and SlCyp1.

Fig. S4. Phloem-expression of *SlCyp1* restores auxin response in *dgt* roots.

## Supplementary Material

supplementary_figures_S1_S4
