# Supplementary material for: Function of Cyclophilin1 as a long-distance signal molecule in the phloem of tomato plants
Source: J Exp Bot. 2017 Jan 4;68(5):953–64. doi: 10.1093/jxb/erw487 (PMC5444435; doi:10.1093/jxb/erw487)
Supplement: supplementary_figures_S1_S4 [file erw487_suppl_supplementary_figures_S1_S4.pdf]

Supplementary Figure 1

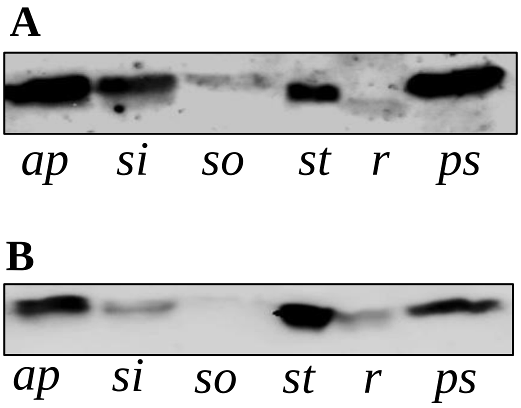

Supplementary figure S1. Spatial distribution of Cyp1 proteins in different organs. Anti-Cyclophilin Western-blot analyses on protein extracts from different plant organs using an anti-AtCyp18-3/ROC1 antiserum. Experiments were performed on samples obtained from cucumber (A) and pumpkin (B). Proteins were extracted from the following organs: shoot apex (*ap*), sink leaf (*si*), source leaf (*so*), stem (*st*), root (*r*), and phloem-sap (*ps*).

Supplementary Figure 2

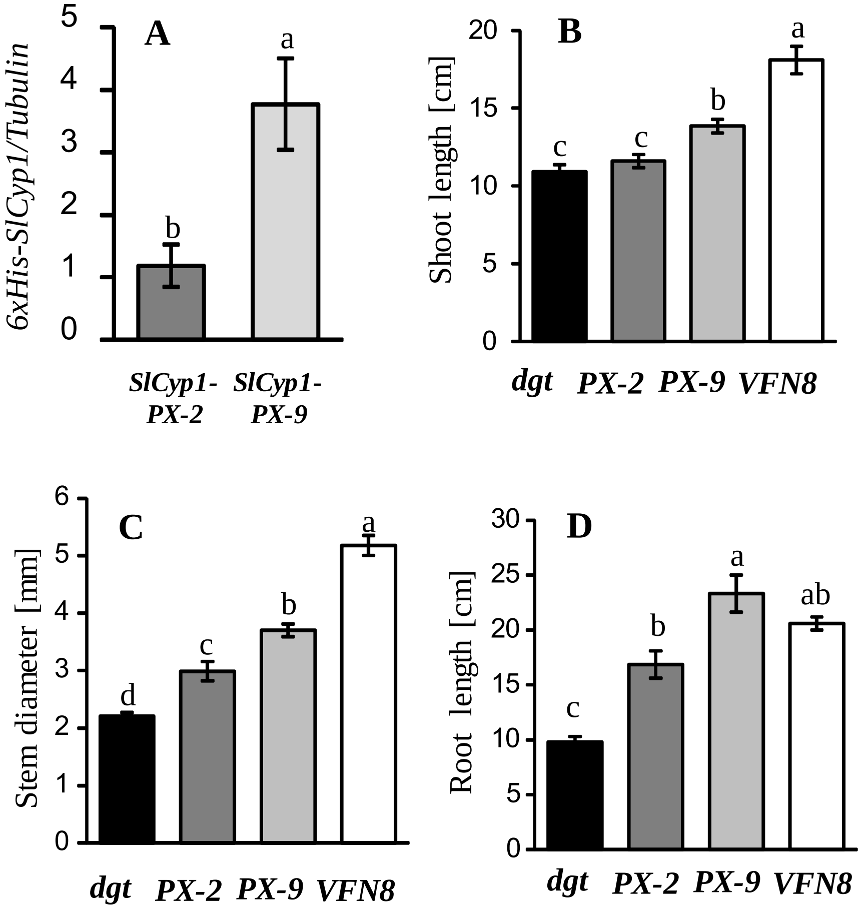

Supplementary figure S2. Effect of phloem-specific expression of *SlCyp1* on shoot and root growth. (A) Relative expression levels of *6xHis-SlCyp1* in stems of two transgenic *dgt* mutant lines expressing *SlCyp1* under the *AtSuc2* promoter (*SlCyp1-PX* lines). Measurements of Shoot length (B), stem diameter (C) and root length (D) of *dgt* mutants (black bars), *SlCyp1-PX-2* (dark grey bars), *SlCyp1-PX-9* (light grey bars) and *VFN8* control plants (empty bars). Data represents means of 5 replications (for A) and 10 replications (for B-D) (±SE). Identical letters indicate no significant differences between genotypes at $p < 0.05$ by Tukey's HSD-test.

.

Supplementary Figure 3

```
HsCypA   1    MVNPTVFFDIAVDGEPLGRVSFELFADKVPKTAENFRALSTGEKGFG-------YKGSCF  53
              M NP VFFD+ + G P GRV  ELFAD  PKTAENFRAL TGEKG G       YKGS F
SlCyp1   1    MANPKVFFDLTIGGAPAGRVVMELFADTTPKTAENFRALCTGEKGVGKMGKPLHYKGSTF  60

HsCypA   54   HRIIPGFMCQGGDFTRHNGTGGKSIYGEKFEDENFILKHTGPGILSMANAGPNTNGSQFF  113
              HR+IPGFMCQGGDFT  NGTGG+SIYG KF DENF+ KHTGPGILSMANAGP TNGSQFF
SlCyp1   61   HRVIPGFMCQGGDFTAGNGTGGESIYGAKFNDENFVKKHTGPGILSMANAGPGTNGSQFF  120

HsCypA   114  ICTAKTEWLDGKHVVFGKVKEGMNIVEAMERFGSRNGKTSKKITIADCGQL  164
              ICTAKTEWL+GKHVVFG+V EGM++++  E  GS +G+ SK + IADCGQL
SlCyp1   121  ICTAKTEWLNGKHVVFGQVVEGMDVIKKAEAVGSSSGRCSKPVVIADCGQL  171
```

<u>Supplementary figure S3. Homology of the human CypA and SlCyp1</u>. Protein alignment of the human cyclophilin A protein (HsCypA) and SlCyp1. Yellow marks the conserved active site residues chosen for site-directed mutagenesis according to Zydowsky *et al.*, 1992.

Supplementary Figure 4

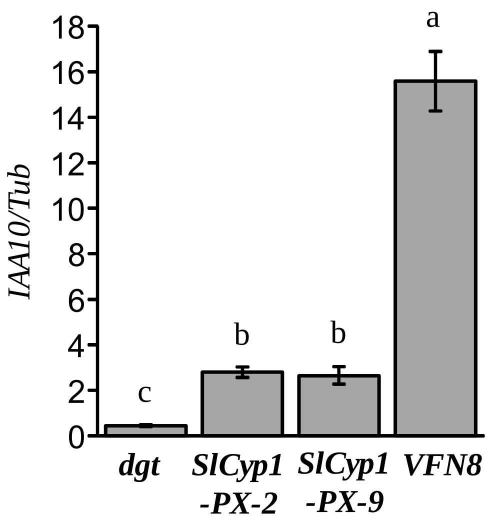

Supplementary figure S4. Phloem-expression of *SlCyp1* restores auxin response in *dgt* roots. Relative transcription level of *IAA10* (*Aux/IAA* family transcript) in roots of *dgt* mutants, SlCyp1-PX-2, SlCyp1-PX-9 and *VFN8* root systems. The indicated data represents means of 6 biological replications ± SE. Identical letters indicate no significant differences between auxin treatments in each genotype at $p < 0.05$ by Tukey's HSD-test.
